# Medication Adherence of People Living with HIV in Japan—A Cross-Sectional Study

**DOI:** 10.3390/healthcare11040451

**Published:** 2023-02-04

**Authors:** Yoji Inoue, Shinichi Oka, Seiji Yokoyama, Koichi Hasegawa, Jörg Mahlich, Ulrike Schaede, Noriyuki Habuka, Yoko Murata

**Affiliations:** 1 Graduate School of Health Care and Nursing, Juntendo University, Chiba 2790023, Japan; 2 AIDS Clinical Center, National Center for Global Health and Medicine, Tokyo 1628655, Japan; 3 Medical Affairs, Janssen Pharmaceutical K. K., Tokyo 1010065, Japan; 4 Health Economics & Outcomes Research, Janssen-Cilag, 41470 Neuss, Germany; 5 DICE, University of Düsseldorf, 40225 Düsseldorf, Germany; 6 School of Global Policy and Strategy, UC San Diego, La Jolla, CA 92093, USA

**Keywords:** Japanese PLHIV, adherence, shared decision-making

## Abstract

Long-term medical care for people living with HIV (PLHIV) is critical for treatment efficacy, and various studies have examined reasons for antiretroviral therapy (ART) non-adherence. In Japan, doctors assume patients maintain high adherence. However, little is known about real-world treatment adherence. We conducted an anonymous self-administered web-based survey asking about adherence for a total of 1030 Japanese PLHIV who were currently on ART. Adherence was determined using the eight-item Morisky Medication Adherence Scale (MMAS-8), for which scoring ranged from 0 to 8 and scores < 6 points were classified as low adherence. Data were analyzed based on patient-related factors; therapy-related factors; condition-related factors, such as a comorbidity with depression (utilizing the Patient Health Questionnaire 9, PHQ-9); and healthcare/system-related factors. Among 821 PLHIV who responded to the survey, 291 responders (35%) were identified as being in the low adherence group. A statistically significant relationship was found between the number of missed anti-HIV drug doses within the previous 2 weeks and long-term adherence, per the MMAS-8 score (*p* < 0.001). Risk factors for low adherence included age (younger than 21 years, *p* = 0.001), moderate to severe depression (*p* = 0.002, using the PHQ-9), and drug dependence (*p* = 0.043). Adherence was also influenced by a shared decision-making process, including treatment selection, doctor–patient relations, and treatment satisfaction. Adherence was mainly affected by treatment decision factors. Hence, support of care providers should be considered critical for improving adherence.

## 1. Introduction

Combination antiretroviral therapy helps prevent the progression of HIV infections to immunodeficiency and has been available since 1996. When initiated promptly and continued with good adherence, the highly active antiretroviral therapy (HAART, now referred to as ART) regimen sustains virological suppression and immunological reconstitution in people living with HIV (PLHIV) and reduces the risk of HIV transmission [1,2]. However, PLHIV still need to continue ART for their entire life and long-term adherence to ART is critical for treatment efficacy. Non-adherence impairs the health prospects of PLHIV and leads to more complicated treatment regimens, which typically have much lower adherence rates than ART. Moreover, non-adherence may lead to antiretroviral resistance, which allows HIV to replicate in patients [3,4,5].

The importance of long-term medical care for PLHIV was first emphasized in 2001 [6]. Subsequently, in 2003, the World Health Organization (WHO) issued the booklet “Adherence to Long-term Therapies: Evidence For Action” [7]. In this booklet, the WHO defines adherence as “the extent to which a person’s behavior (taking medication, following a diet, and/or executing lifestyle changes) corresponds with agreed recommendations from a healthcare provider”. The booklet also discusses five dimensions that should be considered for improving adherence: patient-related factors, therapy-related factors, condition-related factors, healthcare team and system-related factors, and social and economic factors. Various studies have examined the causes of non-adherence in treatment. In a review of 29 articles, Altice et al. [8] found that single-tablet regimens (STRs) had higher adherence than multiple-tablet regimens (MTRs). In a review of 125 studies ranging from 1997 to 2016, Shubber et al. [9] identified the following contributing factors to non-adherence in adult PLHIV: depression, substance and alcohol abuse, forgetfulness, and being away from home. Social factors such as secrecy and stigma have also been suggested as triggers of non-adherence, as have low health literacy and doctor–patient relationships [10]. Moreover, Clucas et al. [11] showed that shared decision-making leads to improved mental and physical conditions of PLHIV and higher levels of adherence. Thus, unhindered good communication between doctors and patients is crucial for the overall health of PLHIV and their adherence to therapy.

By the end of 2019, the cumulative number of reported HIV cases in Japan was 32,825, which is extremely low compared to other countries in terms of prevalence. The Japanese government reimburses the majority of ART costs when it is doctor-prescribed and combined with a recommendation based on the following system. Japanese HIV-positive peoples are generally issued a Physical Disability Certificate, which allows them to take advantage of a system called “medical care for services and supports for persons with disabilities”. Under this system, they only pay a co-payment of up to 20,000 yen per calendar month, based on their previous year’s income. Considering this context, relatively few studies have been conducted in Japan to understand the real-world situation regarding adherence, i.e., whether people are actually taking anti-HIV drugs. Doctors, pharmacists, and nurses are the health professionals who provide support for improving adherence, but only doctors prescribe anti-HIV drugs and they assume that patients maintain high adherence. There is an urgent need to determine the current real-world ART adherence rate in PLHIV and identify factors that may affect adherence to be able to implement measures that can improve future adherence in Japan.

Considering the above, the objectives of this study were to determine real-world adherence to ART among Japanese PLHIV and identify factors associated with ART adherence among PLHIV.

## 2. Materials and Methods

### 2.1. Subjects and Survey Methods

We conducted an anonymous cross-sectional study using a web survey of Japanese PLHIV that were currently using anti-HIV drugs. The cross-sectional study was designed to be anonymous and a self-administered web survey, which seemed more suitable than a paper-based survey, owing to the strong stigma reported among PLHIV [12]. We recruited survey participants by posting banner advertisements in cooperation with PLHIV support groups, the Japanese Network of People living with HIV (Tokyo, Japan), the HIV Futures Japan Project (Tokyo, Japan), a general information website for PLHIV, and an online dating website for men who have sex with men (MSM). As a reward for their cooperation in the survey, we offered electronic gift certificates worth 1000 yen to 300 participants, who were randomly selected in a draw.

The web survey structure allowed participants to first read an explanation about the survey, provide their consent to participate, and then answer the questions. The survey was available in the Japanese language only and consisted of 11 pages and 36 items (see Supplementary Material S1). Duplication of responses from the same respondent was avoided by making further surveys unavailable from the same browser on the same device, and only one response could be submitted from the same terminal and the same browser. The survey period was from 1 April to 31 May 2019.

### 2.2. Variables

We measured adherence using the 8-item Morisky Medication Adherence Scale (MMAS-8). We also measured and determined related factors as variables for analyses and examination. The WHO’s five dimensions of factors affecting adherence were used as reference, although we focused on only four dimensions because the fifth dimension on social and economic factors is more appropriate for developing countries [7]. Data analysis was performed using the following variables: (A) patient-related factors, which included the most common attributes and characteristics studied worldwide; (B) therapy-related factors, which included the number of daily doses and drug tablets; (C) condition-related factors, which included psychological health conditions; and (D) healthcare team and system-related factors to consider when selecting a medication or during discussions with doctors. Each variable is described below.

#### 2.2.1. Adherence

- MMAS-8

The primary outcome variable, medication adherence, was measured using the Japanese version [13] of the MMAS-8. The MMAS-8 was developed by Morisky [14] and originally targeted oral medication for hypertensive patients. The MMAS-8 is now also applied to measure medication adherence in Japan [15,16] and also used in the field of HIV research [17,18].

The MMAS-8 scoring ranged from 0 to 8—8 points equated to high adherence, ≥6 to <8 points as medium adherence, and <6 points as low adherence [14]. In this study, we classified the PLHIV respondents into two groups: respondents with scores ≥ 6 points were classified as medium/high adherence, and respondents with scores < 6 points were classified as low adherence for the purpose of clarifying causes of low adherence. For analysis, if there was only one missing item, then the mean of the sum of the other 7 items was used for the missing item, and the total score was calculated.

- Number of missed doses of anti-HIV drugs

The survey asked PLHIV respondents how many doses of anti-HIV drugs they missed within the last two weeks to verify the MMAS-8 scores.

#### 2.2.2. Influential Factors Affecting Adherence

(A) Patient-related factors

The survey asked questions about socio-demographic variables such as gender, age, sexuality, and final education.

(B) Therapy-related factors

The survey asked the PLHIV respondents to choose their current treatment from the list of response options, including number of daily doses and drug tablets.

(C) Condition-related factors

- Patient Health Questionnaire 9 (PHQ-9)

As for psychological health conditions, we used the Japanese version of the PHQ-9 to assess both the presence and severity of depression symptoms [19,20]. PHQ-9 comprises 9 items for depression treatment according to the United Kingdom’s 2018 National Institute of Health and Clinical Excellence (NICE) guidelines [21]. The American Psychiatric Association (APA) [22] also recommended in 2013 that these 9 items could be used as an evaluation scale for depression [19,20]. We calculated a total score that ranged from 0–27 points and used it to assess symptoms as follows: none, 0–4; mild, 5–9; moderate, 10–14; moderate to severe, 15–19; severe, 20–27.

- Drug dependence (self-reported)

Using the two-case method, PLHIV respondents were asked if drug addiction applied to them.

(D) Healthcare team and system-related factors

In this study, we defined the healthcare team and system-related factors as decision-making factors for treatment and treatment satisfaction. We created variables from the following four aspects: main factors of treatment selection, doctor–patient discussion frequency during treatment selection, information sources used for treatment selection, and treatment satisfaction level.

- Factors involved in treatment selection decision-making

The survey asked PLHIV respondents to select the items they considered important when selecting a medication. These items included drug reputation, dosing frequency, and co-payment amounts.

- Doctor–patient discussions about treatment selection decision-making factors

The survey asked PLHIV respondents about the frequency that they discussed with their doctor when selecting medication.

- Information sources accessed for HIV therapeutic drug selection

The survey asked the PLHIV respondents if they considered doctors as an information source when deciding on HIV therapeutic drugs.

- Treatment satisfaction

The survey asked the PLHIV respondents about their current treatment satisfaction level using a 7-point scale that ranged from “I agree (6 points)” to “I do not agree (0 points)”. We classified the respondents into two groups: high satisfaction (6 points) and low satisfaction (≤5 points).

### 2.3. Statistical Analysis

Survey participants who took less than five minutes to complete their responses were excluded from the analysis. A descriptive analysis involving simple tabulation and calculation of the mean and standard deviation was conducted for each variable. A chi-square test was used for testing independence. For expected frequency values of <5 that exceeded 20%, we used Fisher’s exact test. We used the “low adherence group” as a dependent variable for logistic regression analysis, as well as the crude odds ratio for each variable in the bivariate analysis. In the subsequent multivariate analysis, variables were determined in a hierarchical manner based on the univariate analysis results and preceding studies. In Model 1, socio-demographics variables and current treatment were included. PHQ-9 and drug addiction were added in Model 2, and in Model 3, decision-making factors regarding treatment and treatment satisfaction were added. Cases were excluded in the multivariate analysis if there were missing values.

Statistical analysis packages, SPSS25.0 (IBM Corporation, Armonk, NY, USA) and R3.4.2, were used for the analysis.

### 2.4. Ethical Consideration

The survey was anonymous; thus, identifiable features, including the respondents’ names, addresses, and contact information, were not recorded. Data were processed statistically with sufficient protection and privacy of personal information so that individuals could not be identified.

Clinical registration ID for this study is UMIN000036197.

## 3. Results

### 3.1. PLHIV Respondents and Target Analysis

We obtained survey answers from 1030 PLHIV respondents. The median survey response time was 11 min; first quartile 7 min and third quartile 15 min. After identifying duplicate or incomplete responses and those with a response time of ≤5 min from the study, we excluded 209 respondents. Thus, 821 respondents were included in this study as valid analysis subjects.

### 3.2. Relationships of the Number of Missed Anti-HIV Drug Doses with the Adherence Groups

From the 821 PLHIV respondents analyzed, the middle/high and low adherence groups included 510 (64.4%) and 282 (35.6%), respectively (Table 1). The mean duration of current anti-HIV medication was 3.9 years, SD 3.8 years, and the median was 3 years.

Regarding the number of missed anti-HIV drug doses within the last 2 weeks, 79.7% of respondents answered none, and 20.3% of respondents answered once or more. In the medium/high adherence group, 93.1% answered no missed anti-HIV drug doses within the last 2 weeks, 4.7% answered once, and 2.2% answered twice or more. In contrast, in the low adherence group 55.3% reported no missed anti-HIV drug doses within the last 2 weeks, 18.8% answered one missed dose, and 25.9% answered two or more. The MMAS-8 score was significantly associated with the number of missed anti-HIV drug doses within 2 weeks (*p* < 0.001).

### 3.3. Influential Factors Associated with Adherence

(A) Patient-related factors

The majority of PLHIV respondents were men (783, 95.4%), with a mean age of 40.5 years (SD = 9.0 years)—38.6% were in their 40s, and 30.9% were in their 30s. Most respondents were either gay or lesbian (638, 77.7%), and 421 respondents (51.3%) were university graduates or higher (Table 2).

The medium/high adherence and low adherence groups were significantly associated with gender (*p* = 0.005), age (*p* = 0.001), sexuality (*p* = 0.003), and educational background (*p* = 0.018).

(B) Therapy-related factors

The self-reported current treatment dose frequencies were as follows: 1 tablet/day (43.5%), ≥2 tablets/day (42.4%), 2 times/day (9.9%), and ≥3 times/day (4.3%). Adherence was significantly associated with current treatment dose frequency (*p* < 0.001) (Table 2).

(C) Condition-related factors

As shown in Table 2, there was a significant difference in PHQ-9 depression symptoms between the medium/high adherence group and the low adherence group (*p* = 0.002). No depression symptoms were reported for 47.7% of the medium/high adherence group and 35.1% of the low adherence group. Severe depression symptoms were reported in 3.2% of the medium/high adherence group and 6.2% of the low adherence group.

The percentages of self-reported drug dependence in the medium/high adherence and low adherence groups were 3.2% and 6.2%, respectively (*p* = 0.043).

(D) Healthcare team and system-related factors (current treatment, treatment decision-making factors, and treatment satisfaction)

When selecting a medication, more respondents in the medium/high adherence group selected dosing frequency as an important factor in decision-making than in the low adherence group (80.4% vs. 70.4%, *p* = 0.001, Table 2). In contrast, more respondents in the low adherence group chose drug reputation (33.0% vs. 24.7%, *p* = 0.011) and co-payment amount (34.4% vs. 26.2%, *p* = 0.014) than those in the medium/high adherence group.

Regarding the effect of frequency of doctor–patient discussions on medication selection, 62.8% of the middle/high adherence group “discussed well with doctors”, while 48.1% in the low adherence group “discussed well with doctors”. The percentage of “sometimes discuss with doctors” and “never discussed with doctors” were 30.8% and 6.4%, respectively, in the medium/high adherence group, and 41.2% and 10.7%, respectively, in the low adherence group (*p* < 0.001).

Regarding the respondents who answered “doctors” as an information source for deciding on HIV therapeutic drugs, the medium/high adherence group had more respondents who answered “doctors” (93.0%) than the low group (87.3%) (*p* = 0.006).

Treatment satisfaction in the middle/high adherence group and the low adherence group were 56.2% and 32.3%, respectively (*p* < 0.001).

### 3.4. Logistic Regression Analysis of the Low Adherence Group

#### 3.4.1. Univariate Analysis (Crude Odds Ratios)

As shown in Table 2, we report the statistically significant crude odds ratios for attributes and characteristics such as women to men (odds ratio (OR): 2.29, 95% CI: 1.14–4.62); respondents in their 30s (0.62, 0.39–0.97), 40s (0.42, 0.27–0.65), and 50s or older (0.49, 0.30–0.83) to respondents that were 20-years old or less; bisexual (0.49, 0.25–0.96) and gay/lesbian (0.36, 0.21–0.64) to heterosexual; high school graduates or below (1.62, 1.16–2.26) to university graduates or above; and current treatment regimens ≥3 times daily (3.22, 1.57–6.61) to 1 tablet once daily.

For PHQ-9 depression, all the groups with symptoms showed significantly higher ORs to the group of “none” (PHQ-9 score 0–4), and the odds ratio increased as the severity of symptoms increased (mild, 1.48, 1.04–2.11; moderate, 1.55, 1.03–2.36; moderate to severe, 2.35, 1.41–3.92; severe, 2.63, 1.30–5.30). The odds ratio of respondents with self-reported drug dependence was 1.99 (1.01–3.92).

The low adherence group was more likely to give the following answers than the middle/high adherence group: drug reputation (1.50, 1.10–2.05) and co-payment amount (1.47, 1.08–2.01) as medication selection factors, “sometimes discussing with doctors” (1.75, 1.29–2.38) and “never discussed with doctors” (2.17, 1.28–3.67) as treatment satisfaction factors, and had low treatment satisfaction (2.69, 1.99–3.63). However, respondents who answered that dosing frequency (0.58, 0.42–0.81) was a treatment selection factor and that doctors were an information source choosing HIV therapeutic drugs (0.51, 0.32–0.83) were rarely in the low adherence group.

#### 3.4.2. Hierarchical Multivariate Analysis (Model 1–3)

As a result of hierarchical multivariate analysis, when PHQ-9 and drug dependence were entered in Model 2 in addition to the independent variables entered in Model 1, the significantly related variables in Model 1 remained mostly the same (see Supplementary Material S1 for full results of Model 1–3). Moreover, as shown in Model 2 in Table 3, there was a significant relationship between adherence and moderate to severe (adjusted odds ratio (aOR): 1.98, 1.15–3.41) and severe PHQ-9 depression symptoms (aOR:2.33, 1.11–4.86). This relationship was also observed between adherence and drug-dependent respondents (aOR:2.12, 1.03–4.38). When decision-making factors about treatment and treatment satisfaction were entered in Model 3, a significant relationship was observed for drug dependence (aOR:2.31, 1.09–4.92), but not for PHQ-9.

## 4. Discussion

### 4.1. Adherence in Japanese PLHIV

We surveyed the adherence to ART among Japanese PLHIV, using MMAS-8 criteria. The survey results revealed that 35.4% of the PLHIV respondents had low adherence. In addition, there was a statistically significant relationship between the number of missed anti-HIV drug doses within the last 2 weeks and ART adherence—approximately half of the low adherence group missed doses within the last 2 weeks of taking the survey. Thus, we confirmed the validity of measuring adherence by MMAS-8 criteria used in this survey.

To the best of our knowledge, our current study is the first to examine ART adherence among PLHIV in Japan using the MMAS-8. According to the results of a web-based survey of HIV-positive patients in Japan conducted by the HIV Futures Japan Project in 2017, 66.2% of the 948 patient respondents on ART did not miss any anti-HIV drug doses in the past month [23,24]. The percentage of patient respondents who missed doses in the above survey was very similar and in line with the percentage of PLHIV respondents in the low adherence group in the present study using MMAS-8 criteria.

### 4.2. Relationship of Adherence and Adherence-Related Factors

As shown in Table 2, all the variables assessed showed the association with low adherence. The low adherence group was characterized by having more females, younger ages, and more heterosexual and bisexual respondents than the medium/high adherence group. These results were consistent with the univariate logistic regression analyses (Crude OR in Table 3). Reports by others also support the high adherence in older age in patients with HIV, while not fully consistent trends for gender or sexuality [25,26].

Lower adherence was observed in respondents who had higher dosing frequency per day; a trend also observed in multiple logistic regression analyses (Table 2 and Table 3). Therefore, to increase adherence among Japanese PLHIV, ART drugs with low dosing frequency should be selected. These results are consistent with previous reports [8].

In Japan, there are many HIV treatment guidelines [27,28], and all of these guidelines prioritize fewer medications. Patients also often tend to choose ARTs that require fewer medications. However, in many cases, doctors who are not familiar with HIV treatment or patients whose viral load has been suppressed for a long period of time continue to use ART with a high dosing frequency. From the perspective of improving adherence, it would be desirable to actively introduce ARTs with fewer medications in these cases.

Psychological health conditions, decision-making treatment factors, and treatment satisfaction were associated with adherence. Analyses were performed with variables of attributes/characteristics and current treatment regimens (Model 2 in Table 3), and as a result, the respondents with moderate, moderate to severe, or severe PHQ-9 depression symptoms had lower adherence than respondents with no symptoms. Drug-dependent respondents also had lower adherence than those without drug dependence.

These results are consistent with those from previous studies in other countries [9,29,30]. Thus, improving depression symptoms, mental health, and drug dependence is important for increasing ART adherence.

### 4.3. Mutual Relationships between Adherence and Psychological Health Conditions, Decision-Making Treatment Factors, and Treatment Satisfaction

When treatment decision factors were included as variables, the significant relationship between the level of depression symptoms and adherence disappeared, as shown in Model 3 in Table 3. The results of the hierarchical analysis suggest that depression symptoms lead to a passive attitude that involves readily accepting information, such as drug reputation, without questioning the information due to a lack of motivation or waiting for a doctor’s instructions, causing subsequent low adherence. Depression symptoms do not directly reduce adherence. However, the worsening of depression symptoms leads to negative attitudes towards ART, other treatment, and relationships with doctors and healthcare professionals, lowering adherence.

A literature search on ART yielded no research articles evaluating how depressive symptoms create passive attitudes, thereby decreasing adherence. Previous studies have found that when depression symptoms worsen, treatment satisfaction and adherence decline in patients with type 2 diabetes mellitus [31]. Depression also affects adherence when patient satisfaction declines in regard to their interactions with doctors [32]. Thus, treatment satisfaction declines in Japanese PLHIV with poor psychological conditions when patients have limited communication with their doctor or are reluctant to communicate, which may then cause lower adherence.

In addition, a similar situation was observed for medication selection factors and adherence. We identified drug reputation as one of the factors that significantly reduce adherence. However, the respondents’ interpretation of reputation is unknown—some might prioritize informal drug information over medical and pharmacological information. To improve adherence in this group, doctors may need to carefully discuss drug information with patients while assessing their psychological condition.

Psychological factors, such as depression and drug dependence, may affect treatment decision-making factors and reduce adherence, therefore, doctor–patient communication may not be enough for information collection, assessment, or medication support. To assess complicated events in each patient and provide more effective support, we determined that it is necessary to enhance the collaboration system for medication support by providing a medical team, including a doctor, nurse, pharmacist, social worker, and counsellor [33]. Therefore, the results of our study showed that the healthcare team and system-related factors considerably affect ART adherence.

It is important that patients are proactive in their ART and treatment choices. It may be worthwhile to create and disseminate educational materials such as booklets, websites, and videos that encourage patients to be proactive in discussions with their doctors about their medical care, and to openly discuss and exchange opinions about ART with their doctors.

### 4.4. Limitations of This Study

In this study, we obtained web-based survey responses from approximately 1000 Japanese PLHIV within the context of strong stigma. Even though we were successful at obtaining a large number of responses, the respondents tended to be younger with higher educational backgrounds that are familiar with information and communication technologies. Although the true adherence rate is unlikely to differ greatly from the results of this survey, we cannot deny the possibility that unknown bias may still be lurking in the web survey. In addition, as this was a self-administered survey, there is a possibility that the respondents may have given desirable answers. Therefore, we plan to conduct a survey using a different method in the future to help minimize this information bias. We also need to incorporate qualitative and quantitative factors into future surveys.

## 5. Conclusions

A web-based adherence survey using MMAS-8 identified low ART adherence among 35% of the 821 PLHIV respondents. Risk factors for decreased adherence included being 20 years old or younger, moderate to severe depression symptoms, and drug dependence. Adherence was also affected by treatment decision-making factors, such as medication selection factors, doctor–patient discussions, and treatment satisfaction. Therefore, we believe that a multi-disciplinary medical team supporting patients on ART may improve adherence.

## Figures and Tables

**Table 1:** Number of missed doses of anti-HIV drugs within 2 weeks.

	Total	Medium/High Adherence Group	Low Adherence Group	*p*-Value ^2^
	n = 792 ^1^	n = 510 (64.4%)	n = 282 (35.6%)	
	n (%)	n (%)	n (%)	
None	631 (79.7)	475 (93.1)	156 (55.3)	<0.001
Once	77 (9.7)	24 (4.7)	53 (18.8)	
≥2 times	84 (10.6)	11 (2.2)	73 (25.9)	

^1^ Twenty-nine out of 821 respondents were excluded as they answered 14 times as the number of times that they forgot to take their medication in the past 2 weeks, which was considered as a misunderstanding of the options. ^2^ χ^2^ test

**Table 2:** Comparison of the medium/high adherence and low adherence groups by various factors.

	Total	Medium/High Adherence Group	Low Adherence Group	*p*-Value ^1^
		n = 530 (64.6%)	n = 291 (35.4%)	
	n (%)	n (%)	n (%)	
<Socio-demographics>				
Gender				
Male	783 (95.4)	514 (97.0)	269 (92.4)	0.005
Female	33 (4.0)	15 (2.8)	18 (6.2)	
Others	5 (0.6)	1 (0.2)	4 (1.4)	
Age				
Mean (SD)	40.5 (9.0)	41.5 (8.7)	38.9 (9.2)	
≤20 s	108 (13.2)	54 (10.2)	54 (18.6)	0.001
30 s	254 (30.9)	157 (29.6)	97 (33.3)	
40 s	317 (38.6)	224 (42.3)	93 (32.0)	
50 s/60 s	142 (17.3)	95 (17.9)	47 (16.2)	
Sexuality				
Heterosexual	54 (6.6)	23 (4.3)	31 (10.7)	0.003
Bisexual	105 (12.8)	63 (11.9)	42 (14.4)	
Gay/Lesbian	638 (77.7)	428 (80.8)	210 (72.2)	
Others	24 (2.9)	16 (3.0)	8 (2.7)	
Educational background ^2^				
High school and below	227 (27.7)	130 (24.6)	97 (33.3)	0.018
Vocational school/junior college	172 (21.0)	111 (21.0)	61 (21.0)	
University and above	421 (51.3)	288 (54.4)	133 (45.7)	
<Current treatment>				
1 tablet once daily	357 (43.5)	234 (44.2)	123 (42.3)	<0.001
≥2 tablets once daily	348 (42.4)	239 (45.1)	109 (37.5)	
Twice daily	81 (9.9)	44 (8.3)	37 (12.7)	
(regardless of the number of tablets)				
Three times daily	35 (4.3)	13 (2.5)	22 (7.6)	
(regardless of the number of tablets)				
<Psychological health condition>				
Depressive symptoms (PHQ-9)				
None (0–4)	355 (43.2)	253 (47.7)	102 (35.1)	0.002
Mild (5–9)	222 (27.0)	139 (26.2)	83 (28.5)	
Moderate (10–14)	135 (16.4)	83 (15.7)	52 (17.9)	
Moderate to severe (15–19)	74 (9.0)	38 (7.2)	36 (12.4)	
Severe (≥20)	35 (4.3)	17 (3.2)	18 (6.2)	
Drug dependence (self-reported) ^3^	35 (4.3)	17 (3.2)	18 (6.2)	0.043
<Decision-making factors about and satisfaction with treatment>				
Factors to consider when selecting a medication (multiple answers)				
Reputation of drugs	227 (27.6)	131 (24.7)	96 (33.0)	0.011
Frequency of dosing	631 (76.9)	426 (80.4)	205 (70.4)	0.001
Amount of copayment	239 (29.1)	139 (26.2)	100 (34.4)	0.014
Discussing with doctors about the factors to consider when selecting a medication				
Discussing well with the doctor	473 (57.6)	333 (62.8)	140 (48.1)	<0.001
Discussing sometimes with the doctor	283 (34.5)	163 (30.8)	120 (41.2)	
Never discussed with the doctor	65 (7.9)	34 (6.4)	31 (10.7)	
Doctors as a source of information to determine HIV medication	747 (91.0)	493 (93.0)	254 (87.3)	0.006
Treatment satisfaction				
Low satisfaction	429 (52.3)	232 (43.8)	197 (67.7)	<0.001
High satisfaction	392 (47.7)	298 (56.2)	94 (32.3)	

^1^ Fisher’s exact test for gender and χ^2^ test for the others. ^2^ No response from 1 subject. ^3^ The number shows the respondents who answered that “they are drug dependent”.

**Table 3:** Results of multiple logistic regression analysis with the low adherence group as a dependent variable.

	Crude Odds Ratio				Model 2 Nagelkerke R^2^ = 0.126				Model 3 Nagelkerke R^2^ = 0.183			
	OR ^1^	(95% CI)		*p*- Value	aOR ^2^	(95% CI)		*p*- Value	aOR ^2^	(95% CI)		*p*- Value
<Socio-demographics>												
Gender												
Male	1.00				1.00				1.00			
Female	2.29	(1.14–4.62)		0.020	0.98	(0.41–2.33)		0.963	0.94	(0.39 – 2.25)		0.883
Others	7.64	(0.85–68.72)		0.070	8.06	(0.73–88.79)		0.088	3.14	(0.30 – 33.04)		0.340
Age												
≤20s	1.00				1.00				1.00			
30s	0.62	(0.39–0.97)		0.038	0.65	(0.39–1.06)		0.086	0.61	(0.37–1.02)		0.062
40s	0.42	(0.27–0.65)		<0.001	0.45	(0.27–0.73)		0.001	0.47	(0.28–0.77)		0.003
50s/60s	0.49	(0.30–0.83)		0.007	0.50	(0.28–0.88)		0.015	0.53	(0.29–0.94)		0.031
Sexuality												
Heterosexual	1.00				1.00				1.00			
Bisexual	0.49	(0.25–0.96)		0.038	0.65	(0.30–1.38)		0.264	0.85	(0.39–1.85)		0.682
Gay/Lesbian	0.36	(0.21–0.64)		<0.001	0.61	(0.31–1.21)		0.158	0.99	(0.48–2.03)		0.978
Others	0.37	(0.14–1.01)		0.053	0.26	(0.09–0.77)		0.016	0.36	(0.12–1.12)		0.078
Educational background ^3^												
High school and below	1.62	(1.16–2.26)		0.005	1.44	(1.01–2.05)		0.045	1.43	(0.99–2.06)		0.055
Vocational school/junior col.	1.19	(0.82–1.73)		0.362	1.11	(0.74–1.66)		0.612	1.04	(0.69–1.59)		0.837
University and above	1.00				1.00				1.00			
<Current treatment>												
1 tablet once daily	1.00				1.00				1.00			
≥2 tablets once daily	0.87	(0.63–1.19)		0.376	0.87	(0.62–1.20)		0.390	0.79	(0.56–1.11)		0.178
Twice daily (regardless of the # of tablets)	1.60	(0.98–2.61)		0.059	1.50	(0.88–2.56)		0.140	1.16	(0.65–2.07)		0.609
Three times daily (regardless of the # of tablets)	3.22	(1.57–6.61)		0.001	3.22	(1.37–7.54)		0.007	2.59	(1.07–6.32)		0.036
<Psychological health condition>												
Depressive symptoms (PHQ-9)												
None (0–4)	1.00				1.00				1.00			
Mild (5–9)	1.48	(1.04–2.11)		0.031	1.35	(0.93–1.97)		0.117	1.12	(0.76–1.66)		0.571
Moderate (10–14)	1.55	(1.03–2.36)		0.038	1.22	(0.78–1.92)		0.375	0.95	(0.59–1.53)		0.838
Moderate to severe (15–19)	2.35	(1.41–3.92)		0.001	1.98	(1.15–3.41)		0.013	1.63	(0.94–2.83)		0.085
Severe (≥20)	2.63	(1.30–5.30)		0.007	2.33	(1.11–4.86)		0.025	1.90	(0.89–4.05)		0.097
Drug dependence (self-reported) ^4^	1.99	(1.01–3.92)		0.047	2.12	(1.03–4.38)		0.042	2.31	(1.09–4.92)		0.029
<Decision-making factors about and satisfaction with treatment>												
Factors to consider when selecting a medication (multiple answers)												
Reputation of drugs ^4^	1.50	(1.10–2.05)		0.011					1.44	(1.02–2.03)		0.037
Frequency of dosing ^4^	0.58	(0.42–0.81)		0.001					0.65	(0.44–0.94)		0.023
Amount of copayment ^4^	1.47	(1.08–2.01)		0.014					1.17	(0.82–1.68)		0.377
Discussing with doctors about the factors to consider when selecting a medication												
Discussing well	1.00								1.00			
Discussing sometimes	1.75	(1.29–2.38)		<0.001					1.46	(1.04–2.05)		0.027
Never discussed	2.17	(1.28–3.67)		0.004					1.21	(0.67–2.21)		0.528
Doctors ^4^ as a source of information to determine HIV medication	0.51	(0.32–0.83)		0.006					0.73	(0.41–1.29)		0.274
Low satisfaction with treatment ^5^	2.69	(1.99–3.63)		<0.001					2.23	(1.59–3.11)		<0.001

^1^ odds ratio ^2^ adjusted odds ratio ^3^ n = 820 with 1 person whose educational background was unknown. The other crude odds ratios were calculated using n = 821. Model 1 and 3 were analyzed with n = 820. ^4^ “Not applicable” was set as a reference category. ^5^ The high satisfaction group was set as a reference category.

## Data Availability

The data presented in this study are available on reasonable request from the corresponding author. The data are not publicly available due to privacy.

## Supplementary Materials

The following supporting information can be downloaded at: https://www.mdpi.com/article/10.3390/healthcare11040451/s1, Material S1: Survey for Adherence to HIV Drugs in Japan and Results of multiple logistic regression analysis with full data for Model 1,2, and 3.
