# Supplementary material for: Medication Adherence of People Living with HIV in Japan—A Cross-Sectional Study
Source: Healthcare (Basel). 2023 Feb 4;11(4):451. doi: 10.3390/healthcare11040451 (PMC9957380; doi:10.3390/healthcare11040451)
Supplement: Supplementary file 1 [file healthcare-11-00451-s001.zip › healthcare-2149688-supplementary.pdf]

Supplementary Material

Table of Contents

1. **Survey for Adherence to HIV Drugs in Japan -** Detailed information and translation of the web survey page 2

2. **Table S1.** Results of multiple logistic regression analysis with the low adherence group as a dependent variable (with full data for Model 1,2, and 3) page 21

Survey for Adherence to HIV Drugs in Japan

-Detailed information and translation of the web survey

■ About this survey

The purpose of this survey is to understand the status of medication adherence among HIV-positive people who live in Japan and are currently taking antiretroviral drugs. We believe that clarifying this situation will be useful for medical institutions to plan appropriate medication support.

The number of people who will participate in this survey is about 300. The survey takes about 20 minutes to complete. After the survey period is over, 300 participants will be given an electronic gift certificate worth 1000 yen by lottery. If the planned number of participants is reached during the survey period, the survey may be closed early.

We look forward to your cooperation.

■ Survey items

This questionnaire consists of total 36 questions.

Some questions are further broken down.

A. Attributes

B. Symptoms and current treatment

C. Current decision-making factors for healthcare

D. Medication adherence

E. Patient's preference and current level of satisfaction

F. HIV treatment and self-assessment diagnosis

G. Lifestyle

■ Survey outline

• Survey population

Individuals who are positive for HIV (AIDS virus) who live in Japan and have been receiving treatment with anti-HIV drugs.

• Period

March 2019 – June 2019 (Plan)

• Survey method

Web-based anonymous self-administered questionnaire. Participants can access the questionnaire screen on the website.

• Personal information

This is an anonymous survey.

• Honorarium

Out of those who completed the questionnaire, 300 participants will be given an electronic gift certificate worth 1000 yen by lottery.

■ Survey sponsor

Janssen Pharmaceutical K.K.

■ Public relations support

Japan Network of People Living with HIV/AIDS

HIV Futures Japan Project

*These organizations are not survey sponsors.

“Explanation of the survey and request for consent to participate”

■ Please carefully read this Information Sheet

We would like to invite you to participate in a study to investigate medication adherence among people who are HIV-positive. This study is called a "cross-sectional study," and a questionnaire survey will be conducted on medication adherence for HIV-positive people living in Japan and taking antiretroviral drugs. We will collect the information in only one survey and includes questions collecting information on various psychological conditions and lifestyle. Before deciding whether or not to participate in this research, please carefully read this Information Sheet and fully understand the details before deciding to participate.

If you agree to participate in this study, please check the Consent column at the end of this Information Sheet. If you have any questions, please contact Accelight Inc., a company that manages this website. However, we will not accept inquiries about the question items themselves to not bias the answers.

This research will be conducted according to the "Declaration of Helsinki," which is universally established to secure patients' rights in medical research on humans and ensure research is carried out in an appropriate manner, and the "Ethical Guidelines for Medical and Health Research Involving Human Subjects,” which was established by the Japanese government.

■ About medical research

Medical research is ultimately aimed at obtaining information that can help improve the quality of life of patients. Medical research in humans (hereinafter, abbreviated as medical research) includes those planned by doctors working at medical institutions and those planned by corporations such as pharmaceutical companies. The research described below is planned and carried out by the pharmaceutical company, Janssen Pharmaceutical K.K.

The research presented here is not a "clinical trial" conducted to obtain approval from the government to manufacture and sell new drugs. It is called “cross-sectional research" and is conducted to collect information about HIV observed in normal medical care in one go.

■ Purpose of this research

Currently, there are various antiretroviral drugs for the treatment of HIV infection, but no clear information on medication adherence in Japan, such as whether the medication is taken correctly every day.

The main purpose of this study is to understand the medication adherence status of HIV-positive people who are receiving oral treatment. Clarifying medication adherence will assist medical institutions to decide appropriate medication support.

■ Survey population

At least 300 people will take part in this research in Japan.

Individuals who meet the following conditions can take part in the research:

(1) People living in Japan with a history of HIV infection and taking oral medication
(2) People who understand this research and who are able to provide consent to participate

■ Details of this research

Please understand the details of the research, as shown below. If you are willing to take part in the research, please check the Consent column. Then start the survey on the internet website. The survey takes about 20 minutes to complete. The survey will be completed when all the items are answered.

Information on this survey will be provided by self-report through the website by the HIV-positive people who participated.

■ Survey method

Web-based, anonymous, self-administered questionnaire. You can have access to the questionnaire screen from this website.

■ Main survey items

Questions about age and gender, questions about HIV status, dosage form and administration method of oral drugs, questions about medication adherence, questions about satisfaction level of medication, and questions about behavior and mental conditions

■ Survey period

March 2019 – June 2019 (Plan). If the planned number of participants was achieved during the survey period, the survey may be prematurely closed.

■ Expected benefits, burden and risks
This is a study to investigate medication adherence among HIV-positive people. Accordingly, there will be no direct benefits nor disadvantages to participating patients.

■ Funding for this research
Funding for this research is provided by Janssen Pharmaceutical K.K.

■ Payment to you as a result of participation in the research
Those who take part will be asked to fill in the questionnaire with data. To reduce the burden associated with data entry, 300 people will receive an electronic e-mail gift of 1,000 yen by lottery after completion of the survey period.
You will be asked to provide an e-mail address to receive the gift certificate once you have completed the survey. Please note that registered e-mail addresses will not be used for purposes other than the gift certificate. The registered e-mail addresses will strictly be retained by the company in charge of this survey, outsourced for this purpose by Janssen Pharmaceutical K.K.

■ Handling and protection of your personal information
Information obtained from you through this study will be used only for the purpose of this study. Personal information that may identify an individual will not be collected.

■ Voluntary participation in the research
It is up to you whether you take part in this study, and participation is completely voluntary. You are not forced to participate in the study. Participation is not forced by anyone, and you do not have to worry about anyone. Your wish to participate or not to participate will be fully respected.

[Consent to participate]
■ Consent Form
Please read carefully before you sign the Consent Form
This Information Sheet and Consent Form contain important information. Please use these as a reference when deciding whether or not to participate in this research. If you have any questions, please contact Accelight Inc., the company providing this website, before participating.

■ Consent for participation in the research
I have read this Information Sheet and understand the details.
Based on the information provided, I voluntarily take part in this research.

Yes / No

(Please check either "Yes" or "No")

→ *(For those who answered "Yes")*
Please go to the next page [Introduction].

→ *(For those who answered "No")*
The following message will be displayed: We are sorry. If you feel like participating at a later date, please re-access this website. We are always waiting for you. Thank you for your response.

Survey for HIV Treatment Information

(Note) Hereafter, option numbers are displayed for single-choice response options, and ☐ is displayed for multiple-choice response options.

[Introduction]

1. Please tell us your residence location. (Mandatory item)

1. Hokkaido
2. Aomori
3. Iwate
4. Miyagi
5. Akita
6. Yamagata
7. Fukushima
8. Ibaraki
9. Tochigi
10. Gunma
11. Saitama
12. Chiba
13. Tokyo
14. Kanagawa
15. Niigata
16. Toyama
17. Ishikawa
18. Fukui
19. Yamanashi
20. Nagano
21. Gifu
22. Shizuoka
23. Aichi
24. Mie
25. Shiga
26. Kyoto
27. Osaka
28. Hyogo
29. Nara
30. Wakayama
31. Tottori
32. Shimane
33. Okayama
34. Hiroshima
35. Yamaguchi
36. Tokushima
37. Kagawa
38. Ehime
39. Kochi
40. Fukuoka

41. Saga
42. Nagasaki
43. Kumamoto
44. Oita
45. Miyazaki
46. Kagoshima
47. Okinawa
48. Overseas

→ *(For those who answered "Overseas")*
The following message will be displayed: "We are sorry. This survey is only for those who currently live in Japan. If there is another opportunity, please participate in the survey."

→ *(For those who answered any response other than "48. Overseas")*

2. Have you ever been diagnosed as HIV-positive? (Mandatory item)

1.Yes
2.No

→ *(For those who answered "2. No")*
The following message will be displayed: "We are sorry. This survey is conducted for people who are HIV-positive. If there is another opportunity, please participate in the survey."

→ *(For those who answered "1. Yes")*

3. Do you regularly visit a clinic and take medication for HIV? (Mandatory item)

1.I am taking medication.
2.I am not taking medication but regularly visiting a clinic.
3.I am not taking medication nor regularly visiting a clinic.

→ *(For those who answered either "2.I am not taking medication but regularly visiting a clinic" or "3. I am not taking medication nor regularly visiting a clinic")*
The following message will be displayed: "We are sorry. This survey is conducted for people who are taking medication and regularly visiting a clinic for HIV. If there is another opportunity, please participate with the survey.

→ *(For those who answered "1. I am taking medication")*

[A. Attributes]

Question 1. What is your gender?

1.Male
2.Female
3.Other

Question 2. What is your age?

____ years old

Question 3. What is your sexual orientation?
(This is a single-choice question, but multiple choices are allowed if "Transgender" was selected)
If you want to change your selected item, please remove a tick first before selecting a new one.

1. Heterosexual
2. Bisexual
3. Gay
4. Lesbian
5. Transgender
6. Other
7. Do not know
8. Do not want to decide

Question 4. What is your highest level of education attained?

1. Middle school
2. High school
3. Vocational school
4. College
5. University
6. Graduate school
7. Other
   Specify: Free-form response

Question 5. What is your main occupation?

1. Company owner / executive
2. Full-time worker (including public servant)
3. Part-time / contracted / commissioned employees / staff
4. Temporary staff
5. Contractor
6. Self-employed / freelancer
7. Family employee
8. Part-time / albeit
9. Housewife / househusband
10. On leave
11. Unemployed

12. Other
    Specify: <u>Free-form response</u>

Question 6. Please tell us the number of other people living with you **(excluding yourself).**

____ people

Question 7. What was the approximate household income (including tax) in 2018? This annual income is not only your income but the total income of all the household members.

1. <1 million yen
2. 1 million yen – 2 million
3. 2 million yen – 3 million
4. 3 million yen – 4 million
5. 4 million yen – 5 million
6. 5 million yen – 6 million
7. 6 million yen – 7 million
8. 7 million yen – 8 million
9. 8 million yen – 9 million
10. 9 million yen – 10 million
11. 10 million yen – 15 million
12. 15 million yen or higher
13. Do not know

Question 8. What was your approximate personal income (including tax) in 2018? This annual income is not your household income, but **your personal income.**

1. <1 million yen
2. 1 million yen – 2 million
3. 2 million yen – 3 million
4. 3 million yen – 4 million
5. 4 million yen – 5 million
6. 5 million yen – 6 million
7. 6 million yen – 7 million
8. 7 million yen – 8 million
9. 8 million yen – 9 million
10. 9 million yen – 10 million
11. 10 million yen – 15 million
12. 15 million yen or higher
13. Do not know

Question 9. Have you ever diagnosed with any of the following diseases or symptoms by a doctor? **Please check all that apply.**

☐ Hypercholesterolemia
☐ Migraine
☐ Diabetes
☐ Hypertension
☐ Obesity
☐ Neurosis

- ☐ Depression
- ☐ Heart disease
- ☐ Nothing applicable

Question 10. Please tell us your current treatment for HIV.

1. 1 tablet, once daily
2. 2 tablets or more, once daily
3. Twice daily (Irrespective of the number of tablets)
4. Three times daily (Irrespective of the number of tablets)
5. Four times daily (Irrespective of the number of tablets)
6. 5 times daily or more

Question 11. How many times do you forget to take HIV drugs a week?

____ times

[B. Symptoms and current treatment]

Question 12. Please provide us with the following numbers.

1) Recent CD4 positive T cell count

1. <200/µl
2. ≥200/µl, <500/µl
3. ≥500/µl
4. Do not know

2) Recent HIV viral load

1. Do not know
2. Never detected
3. Detected at least once in the most recent 3 tests
   Its level is:
   1. <200 copies/ml
   2. ≥200 copies/ml, <1000 copies/ml
   3. ≥1000 copies/ml

3) Height (To the nearest cm)

____ cm

4) Bodyweight (To the nearest kg)

____ kg

Question 13. Do you currently smoke? If your answer is “Yes,” how many cigarettes do you smoke a day?

1. Yes, I currently smoke.
   Number of cigarettes: Approximately ____/day
2. No, I used to smoke before, but am a non-smoker now.
3. No, I have never smoked.

Question 14. Do you currently drink? If your answer is “Yes,” how many glasses of alcohol do you drink a day?
*One (1) glass of alcohol is equivalent to “1 bottle of beer (500 ml),” “1 bottle of Japanese sake (180 ml),” “1 glass of whiskey double (60 ml),” “0.6 bottle of Shochu (110 ml),” “1 glass of wine (180 ml),” “1.5 can of Chuhai (520 ml).

1. Yes, I currently drink.
   How much do you drink: Average ____ glass/day
2. No, I used to drink before, but I do not drink now.
3. No, I have never drunk alcohol.

Question 15. Please select all that apply to you.

☐ Nicotine dependency
☐ Drug abuse

- ☐ Alcohol addiction
- ☐ Shopping addiction
- ☐ Sex addiction / sexual obsessive-compulsive disorder
- ☐ Eating disorder (Overeating, anorexia)
- ☐ Interpersonal addiction
- ☐ Self-harm
- ☐ Others
  Specify: *Free-form response*
- ☐ None of the above

Question 16. The following is written about anti-HIV medications and the experience of medication in the **last 4 weeks**. For each sentence, please select only one response that most closely matches your feelings.

		I agree						I do not agree
1)	I am satisfied with the current treatment.	6	5	4	3	2	1	0
2)	HIV is well under control recently.	6	5	4	3	2	1	0
3)	I am satisfied with the nature of side effects and their severity due to my current treatment.	6	5	4	3	2	1	0
4)	My current treatment feels demanding in terms of time, effort, and thinking.	6	5	4	3	2	1	0
5)	Recent HIV treatment is good for me.	6	5	4	3	2	1	0
6)	Recent HIV treatment is flexible.	6	5	4	3	2	1	0
7)	I fully understand my HIV treatment.	6	5	4	3	2	1	0
8)	I am satisfied that HIV treatment fits with my lifestyle.	6	5	4	3	2	1	0
9)	I want to recommend this treatment combination to other HIV patients.	6	5	4	3	2	1	0
10)	I am satisfied with the continuation of the current treatment.	6	5	4	3	2	1	0

Question 17. How many times was your HIV medication changed in the past?

1. Never
2. Once
3. Twice
4. 3 times
5. 4 times or more

Question 18. Among the reasons that resulted in the switch of HIV drugs recently, please select the one that is most applicable.

1. Due to a side-effect
2. Due to interactions among multiple drugs
3. Due to insufficient efficacy
4. Due to lack of sufficient communication with a doctor
5. Recommendation by a doctor
6. Lifting of diet restriction by changing medications

7. Due to the high frequency of oral medication
8. Due to too many oral medications
9. Due to large tablet size
10. Others
    Specify: *Free-form response*
11. No specific reason

Question 19. How long have you been taking your current HIV medication.
If it is less than 1 year, please enter 0.

0. 0 years
1. 1 year
2. 2 years
3. 3 years
4. 4 years
5. 5 years
6. 6 years
7. 7 years
8. 8 years
9. 9 years
10. 10 years
11. 11 years
12. 12 years
13. 13 years
14. 14 years
15. 15 years
16. 16 years
17. 17 years
18. 18 years
19. 19 years
20. 20 years
21. 21 years
22. 22 years
23. 23 years
24. 24 years
25. 25 years
26. 26 years
27. 27 years
28. 28 years
29. 29 years
30. 30 years
31. 31 years
32. 32 years
33. 33 years
34. 34 years

[C. Current decision-making factors for healthcare]

Question 20. Please select **all that apply** when considering the selection of HIV drugs.

- ☐ Drug's efficacy (e.g., strong inhibition of virus, less occurrence of drug resistance)
- ☐ Drug reputation
- ☐ Drug safety (Fewer gastrointestinal side effects)
- ☐ Frequency of medication (e.g., once daily, twice daily, 3 times daily, or more frequent)
- ☐ No. of tablets per dose (e.g., 1 tablet, 2 tablets, or 3 or more tablets at each medication dose)
- ☐ Conditions for medication (e.g., taken together with meal, 30 minutes after meal)
- ☐ Recommendation by a doctor
- ☐ Information from other sources (e.g., Internet, magazines)
- ☐ Drug price
- ☐ Co-payment amount
- ☐ Other financial support for co-payment (e.g., amount reduced from co-payment, high-cost medical expenses, benefit system)
- ☐ Others

  Specify: *Free-form response*

Question 21. Please select the **top 2 factors** that are taken into consideration when selecting HIV drugs.
(Only items selected at Question 20 are displayed)

- ☐ Drug's efficacy
- ☐ Drug reputation
- ☐ Drug safety (e.g., strong inhibition of virus, less occurrence of drug resistance, less gastrointestinal side effects)
- ☐ Frequency of medication (e.g., once daily, twice daily, 3 times daily, or more frequent)
- ☐ No. of tables per dose (e.g., 1 tablet, 2 tablets or 3 tablets or more at each medication)
- ☐ Conditions for medication e.g., (taken together with meal, 30 minutes after meal)
- ☐ Recommendation by a doctor
- ☐ Information from other sources (e.g., Internet, magazines)
- ☐ Drug price
- ☐ Co-payment amount
- ☐ Other financial support for co-payment (e.g., amount reduced from co-payment, high-cost medical expenses, benefit system)
- ☐ Others

  Specify: *Free-form response*

Question 22. Did you discuss these factors with your doctor?

1. It was well discussed with a doctor.
2. I have sometimes discussed these with a doctor.
3. I have never discussed these factors with a doctor.

Question 23. Where do you obtain information to select HIV drugs? Please check **all that apply.**

- ☐ Doctor
- ☐ Pharmacist
- ☐ Nurse
- ☐ Family / friend
- ☐ Internet
- ☐ Leaflet

- ☐ Other

  Specify: *Free-form response*
- ☐ I am not actively looking for information.

[D. Medication adherence]

Question 24. You answered that you are taking HIV drug(s). We are questioning many people about issues related to the use of HIV drugs. We also want to obtain your experiences. There is no correct answers nor wrong answers to this question. Please share your experiences with HIV drugs with us.

		Yes	No
1	Do you sometimes forget to take your pills?	1	2
2	People sometimes miss taking their medications for reasons other than forgetting. Thinking over the past two weeks, were there any days when you did not take your medicine?	1	2
3	Have you ever cut back or stopped taking your medicine without telling your doctor because you felt worse when you took it?	1	2
4	When you travel or leave home, do you sometimes forget to bring along your medicine?	1	2
5	Did you take all your medicine yesterday?	1	2
6	When you feel like your symptoms are under control, do you sometimes stop taking your medicine?	1	2
7	Taking medicine every day is a real inconvenience for some people. Do you ever feel hassled about sticking to your treatment plan?	1	2

Question 25. How often do you have difficulty remembering to take all your medicine?

1. Never/rarely
2. Once in a while
3. Sometimes
4. Usually
5. All the time

Question 26. Over the **last 2 weeks**, how often have you been bothered by any of the following problems?

		Not at all	Several days	More than half the days	Nearly every day
1	Little interest or pleasure in doing things	1	2	3	4
2	Feeling down, depressed, or hopeless	1	2	3	4
3	Trouble falling or staying asleep, or sleeping too much	1	2	3	4
4	Feeling tired or having little energy	1	2	3	4
5	Poor appetite or overeating	1	2	3	4
6	Feeling bad about yourself — or that you are a failure or have let yourself or your family down	1	2	3	4
7	Trouble concentrating on things, such as reading the newspaper or watching television	1	2	3	4
8	Moving or speaking so slowly that other people could have noticed? Or the opposite — being so fidgety or restless that you have been moving around a lot more than usual	1	2	3	4
9	Thoughts that you would be better off dead or of hurting yourself in some way	1	2	3	4

[E. Patient’s preference and current level of satisfaction]

Question 28. How important is it for you to take part in the selection process of treatment strategy (e.g., to express your opinions, to make proposals)?

1. Very important
2. Somewhat important
3. Not so important
4. Not important at all

Question 29. This is a question related to Question 28. When you select treatment strategy, does your doctor listen to your opinions or proposal? How well do they listen?

1. Listens to me quite a lot
2. Somewhat listens to me
3. Does not listen to me so much
4. Does not listen to me at all
5. I have never expressed my opinions or proposals myself.

Question 30. This is a question related to Question 29. How satisfied are you with that? Please select only one answer.

1. Very satisfied
2. Somewhat satisfied
3. Not very satisfied
4. Not satisfied at all

[F. HIV treatment and self-assessment diagnosis]

Question 31. For each sentence, please select the response closest to your opinions.

		Fully agree	Agree	Likely to agree	Do not think so	Do not think	Not at all
1)	It is good for my health to receive a periodic medical HIV review.	1	2	3	4	5	6
2)	My HIV treatment plan is very helpful to keep my HIV infection under control.	1	2	3	4	5	6
3)	HIV drugs are useful to suppress HIV.	1	2	3	4	5	6
4)	Once HIV drugs are prescribed, it is important to take them every day to control the HIV infection.	1	2	3	4	5	6
5)	If I do not take HIV drugs every day, it negatively affects HIV infection control.	1	2	3	4	5	6
6)	Even for HIV-positive patients, if their health condition is good, it is safe to stop taking HIV drugs.	1	2	3	4	5	6
7)	Even for HIV-positive people, if we follow the recommended HIV treatment, we can live longer.	1	2	3	4	5	6
8)	For HIV infection control, there are many things that we can do ourselves.	1	2	3	4	5	6
9)	My behavior may affect HIV infection in both directions, good and bad.	1	2	3	4	5	6
10)	My behavior does not affect the future of my HIV infection.	1	2	3	4	5	6

Question 32. For each sentence, please select the response closest to your opinion.

		Very applicable	Applicable	Somewhat applicable	Not so much applicable	Not applicable	Not at all
1	I know the daily life of people positive for HIV.	1	2	3	4	5	6
2	I have good knowledge about how HIV is transmitted from person to person.	1	2	3	4	5	6
3	I know a lot about medication for HIV infection.	1	2	3	4	5	6
4	I have a lot of knowledge about the side effects of HIV medication.	1	2	3	4	5	6

Question 33. How confident are you in your knowledge about medical information and treatment of HIV?

1. Very confident
2. Fairly confident
3. Somewhat confident
4. Not very confident
5. Not confident at all

[G. Lifestyle]

Question 34. From now on, you will be asked about exercises in the last 7 days and your “exercise plan” for the coming 7 days. “Exercise” includes aerobic exercise, muscle training, daily activities (e.g., using stairs rather than the elevator).

1) How many days did you exercise in the last 7 days?

0. 0 days (No exercise)
1. 1 day
2. 2 days
3. 3 days
4. 4 days
5. 5 days
6. 6 days
7. Everyday

2) Please tell us how applicable the following sentences are to you.

		Applicable				Not applicable		
1	I want to do exercise in the coming 7 days.	1	2	3	4	5	6	7
2	I have already prepared an exercise plan for the coming 7 days.	1	2	3	4	5	6	7
3	My family, friends, and healthcare professionals anticipate that I will exercise in the coming 7 days.	1	2	3	4	5	6	7
4	I will, in principle, decide myself if I will exercise in the coming 7 days.	1	2	3	4	5	6	7

Question 35. To what extent have the following events occurred in **the last 4 weeks**?

		Very much	Somewhat	Difficult to say	Not so much	Not at all
1	My health condition affected the daily relationship with my family, friends, neighbors, and other people.	1	2	3	4	5
2	My health condition affected my hobbies and leisure activities.	1	2	3	4	5
3	My health condition affected housework.	1	2	3	4	5
4	My health condition affected daily activities or shopping.	1	2	3	4	5

Question 36. Please tell us the level of your awareness of the following activities.

1) Frequency of meals in a day	1-2 meals	3 meals	Often 4 meals or more

2) Attention to calorie uptake and nutritional balance	Always conscious	Am sometimes conscious	Not conscious at all

3) How often did you eat out in the last 7 days? Please tell us the number of times.
____ times

[End of the survey]

All questions were completed.

If you want to end the survey, please click the button below.

Table S1. Results of multiple logistic regression analysis with the low adherence group as a dependent variable (with full data for Model 1,2, and 3)

	Crude odds ratio				Model 1				Model 2				Model 3			
					Nagelkerke $R2 = 0.099$				Nagelkerke $R2 = 0.126$				Nagelkerke $R2 = 0.183$			
	Odds ratio	95% confidence Lower limit	Upper limit	*p* -value	Adjusted Odds ratio	95% confidence Lower limit	Upper limit	*p* -value	Adjusted Odds ratio	95% confidence Lower limit	Upper limit	*p* -value	Adjusted Odds ratio	95% confidence Lower limit	Upper limit	*p* -value
**< Socio-demographics >**																
Gender																
Male	1.00				1.00				1.00				1.00			
Female	2.29	1.14	4.62	0.020	1.00	0.43	2.35	0.995	0.98	0.41	2.33	0.963	0.94	0.39	2.25	0.883
Others	7.64	0.85	68.72	0.070	8.09	0.78	83.66	0.080	8.06	0.73	88.79	0.088	3.14	0.30	33.04	0.340
Age																
≤ 20s	1.00				1.00				1.00				1.00			
30s	0.62	0.39	0.97	0.038	0.63	0.39	1.03	0.066	0.65	0.39	1.06	0.086	0.61	0.37	1.02	0.062
40s	0.42	0.27	0.65	<0.001	0.44	0.27	0.71	0.001	0.45	0.27	0.73	0.001	0.47	0.28	0.77	0.003
50s/60s	0.49	0.30	0.83	0.007	0.45	0.26	0.79	0.005	0.50	0.28	0.88	0.015	0.53	0.29	0.94	0.031
Sexuality																
Heterosexual	1.00				1.00				1.00				1.00			
Bisexual	0.49	0.25	0.96	0.038	0.76	0.36	1.59	0.461	0.65	0.30	1.38	0.264	0.85	0.39	1.85	0.682
Gay/Lesbian	0.36	0.21	0.64	<0.001	0.68	0.35	1.35	0.273	0.61	0.31	1.21	0.158	0.99	0.48	2.03	0.978
Others	0.37	0.14	1.01	0.053	0.32	0.11	0.96	0.042	0.26	0.09	0.77	0.016	0.36	0.12	1.12	0.078
Educational background[1]																
High school and below	1.62	1.16	2.26	0.005	1.53	1.08	2.16	0.017	1.44	1.01	2.05	0.045	1.43	0.99	2.06	0.055
Vocational school/junior college	1.19	0.82	1.73	0.362	1.10	0.74	1.64	0.640	1.11	0.74	1.66	0.612	1.04	0.69	1.59	0.837
University and above	1.00				1.00				1.00				1.00			
**< Current treatment >**																
1 tablet once daily	1.00												1.00			
≥ 2 tablets once daily	0.87	0.63	1.19	0.376	0.87	0.63	1.21	0.406	0.87	0.62	1.20	0.390	0.79	0.56	1.11	0.178
Twice daily (regardless of the number of tablets)	1.60	0.98	2.61	0.059	1.59	0.94	2.70	0.084	1.50	0.88	2.56	0.140	1.16	0.65	2.07	0.609
Three times daily (regardless of the number of tablets)	3.22	1.57	6.61	0.001	3.31	1.44	7.64	0.005	3.22	1.37	7.54	0.007	2.59	1.07	6.32	0.036
**< Psychological health condition >**																
PHQ-9																
None (0-4)	1.00								1.00				1.00			
Mild (5-9)	1.48	1.04	2.11	0.031					1.35	0.93	1.97	0.117	1.12	0.76	1.66	0.571
Moderate (10-14)	1.55	1.03	2.36	0.038					1.22	0.78	1.92	0.375	0.95	0.59	1.53	0.838
Moderate to severe (15-19)	2.35	1.41	3.92	0.001					1.98	1.15	3.41	0.013	1.63	0.94	2.83	0.085
Severe (≥ 20)	2.63	1.30	5.30	0.007					2.33	1.11	4.86	0.025	1.90	0.89	4.05	0.097
Drug dependence (self-reported)[2]	1.99	1.01	3.92	0.047					2.12	1.03	4.38	0.042	2.31	1.09	4.92	0.029
**< Decision-making factors about and satisfaction with treatment >**																
Factors to consider when selecting a medication																
Reputation of drugs[2]	1.50	1.10	2.05	0.011									1.44	1.02	2.03	0.037
Frequency of dosing[2]	0.58	0.42	0.81	0.001									0.65	0.44	0.94	0.023
Amount of copayment[2]	1.47	1.08	2.01	0.014									1.17	0.82	1.68	0.377
Discussing with doctors about the factors to consider when selecting a medication																
Discussing well with the doctor	1.00												1.00			
Discussing sometimes with the doctor	1.75	1.29	2.38	<0.001									1.46	1.04	2.05	0.027
Never discussed with the doctor	2.17	1.28	3.67	0.004									1.21	0.67	2.21	0.528
Doctors as a source of information to determine HIV medication	0.51	0.32	0.83	0.006									0.73	0.41	1.29	0.274
Group of low satisfaction with treatment[3]	2.69	1.99	3.63	<0.001									2.23	1.59	3.11	<0.001

[1] n = 820 with 1 person whose educational background is unknown. The other crude odds ratios were calculated using n = 821. Model 1, 2, and 3 were analyzed with n = 820.

[2] "Not applicable" was set as a reference category.

[3] The high satisfaction group was set as a reference category.
